# STEMI Antithrombotic Therapy: The Evolving Role of P2Y12 Inhibitor Pretreatment in Contemporary Practice

**DOI:** 10.31083/j.rcm2511416

**Published:** 2024-11-21

**Authors:** Antonella Tommasino, Vincenzo Fiorentini, Giulia Mattaroccia, Alessandra Scoccia, Emanuele Barbato

**Affiliations:** ^1^Cardiology Unit, Sant'Andrea University Hospital, 00189 Rome, Italy; ^2^Department of Clinical and Molecular Medicine, Sapienza University of Rome, 00189 Rome, Italy

**Keywords:** acute coronary syndrome, ST-elevation myocardial infarction, pretreatment, P2Y12 inhibitors

## Abstract

The P2Y12 receptor plays a central role in platelet activation, secretion, and procoagulant activity. The CURE (clopidogrel in unstable angina to prevent recurrent events) trial, conducted in 2001, was the first to effectively demonstrate the benefit of dual anti-aggregation therapy with aspirin and clopidogrel in patients with acute coronary syndromes (ACS) undergoing invasive treatment. Since then, the field of interventional cardiology has changed considerably. The introduction of drug-eluting stents (DES) and the development of new, potent P2Y12 inhibitors such as ticagrelor, prasugrel and cangrelor have revolutionized the treatment of ACS. Nevertheless, ST-elevation myocardial infarction (STEMI) remains a critical condition that requires rapid and effective intervention. The use of P2Y12 receptor antagonists as part of the pretreatment strategy is an interesting topic to optimize outcomes in STEMI patients. This review summarizes the existing evidence on the efficacy and safety of pretreatment with P2Y12 receptor antagonists in STEMI, and emphasizes the importance of making pretreatment decisions based on individual clinical characteristics. The review also looks to the future, pointing to the potential role of artificial intelligence (AI) in improving STEMI diagnosis and treatment decisions, suggesting a future where technology could improve the accuracy and timeliness of care for STEMI patients.

## 1. Introduction 

Cardiovascular disease (CVD) remains the primary cause of mortality and morbidity worldwide, with acute coronary syndrome (ACS) being the most common first clinical manifestation [1, 2, 3, 4]. Although the incidence rate of ST-elevation myocardial infarction (STEMI) has decreased, the mortality rate is still approximately 10% [5]. The primary cause of STEMI is acute coronary thrombosis, which results from the rupture, erosion, or dissection of an atherosclerotic plaque. The steps in the development of the thrombus are: (1) platelet adhesion to the arterial wall; (2) platelet activation; (3) release of granules from platelets leading to additional activation; (4) release of tissue factors triggering the initiation of the coagulation process.

Platelets play a key role in adhesion to the site of injury in the arterial wall. This process is triggered when the endothelial surface of the artery is damaged or injured and the underlying collagen is exposed. The platelets then bind to the collagen via specific receptors, such as the platelet glycoprotein VI (GPVI) receptor, facilitating the initial adhesion [6].

In particular, the GPVI and integrin α2β1 (VLA-2, GPIa/IIa) receptors bind directly to the subendothelial collagen, while the combination of GPIb-IX-V and integrin αIIbβ3 (GPIIb/IIIa) binds to the von Willebrand factor (vWF) immobilized by collagen [7, 8, 9].

As soon as the platelets adhere to the damaged arterial wall, they are activated. During activation, the platelets change their shape and receptor expression and release soluble mediators such as adenosine diphosphate (ADP), thromboxane A2 (TXA2) and thrombin [10]. The activation of the P2Y12 receptor by ADP leads to the inhibition of adenylyl cyclase (AC) and thus to platelet aggregation. At the same time, activation of the P13 kinase (P13K) leads to activation of the fibrinogen receptor. Fibrinogen plays a decisive role in this process, as it acts a bridge between the activated GPIIb/IIIa on the platelet surface forming the thrombus.

In addition, platelets may play a crucial role in ischemic reperfusion injury by contributing to microvascular obstruction resulting from microthrombus formation, increased platelet-leukocyte aggregation and the release of potent vasoconstrictor and pro-inflammatory molecules [11]. Microvascular obstruction can manifest as a phenomenon of absent or slow blood flow and is a prognostic factor related to the extent of the infarcted area [12].

For these reasons, drugs that inhibit platelet activity have become the mainstay of STEMI treatment. To date, the standard therapy is dual antiplatelet therapy (DAPT), which consists of aspirin and a P2Y12 receptor antagonist.

However, while aspirin should be administered immediately after the diagnosis of STEMI, there is uncertainty about the optimal timing for the administration of the P2Y12 receptor antagonist [2].

## 2. Oral P2Y12 Inhibitors 

**Fig. 1.**
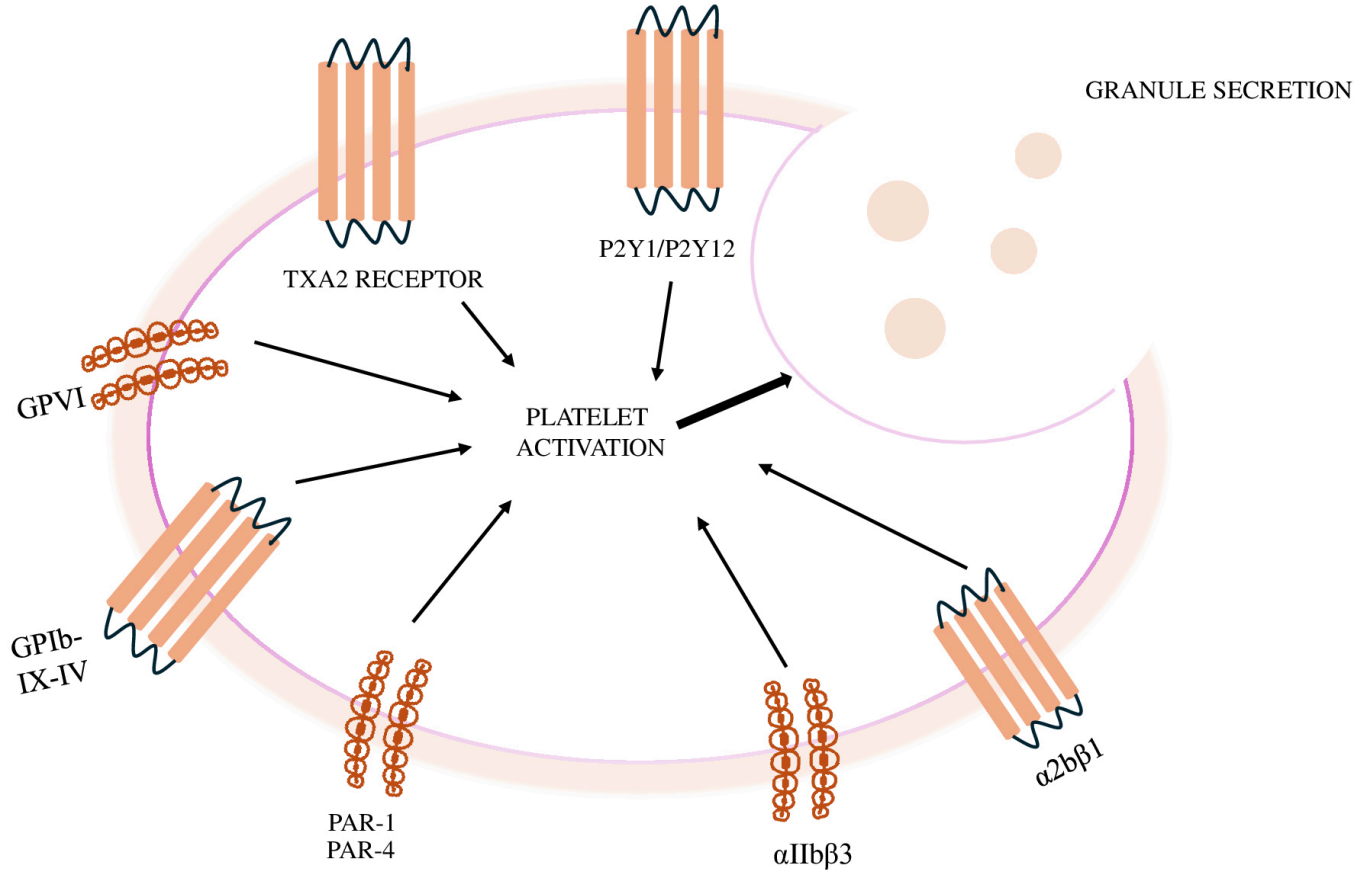
**Mechanisms of platelet activation**. GPVI, platelet glycoprotein VI; TXA2, thromboxane A2; PAR, protease activate receptor.

Fig. 1 summarises the mechanisms of platelet activation.

**Fig. 2.**
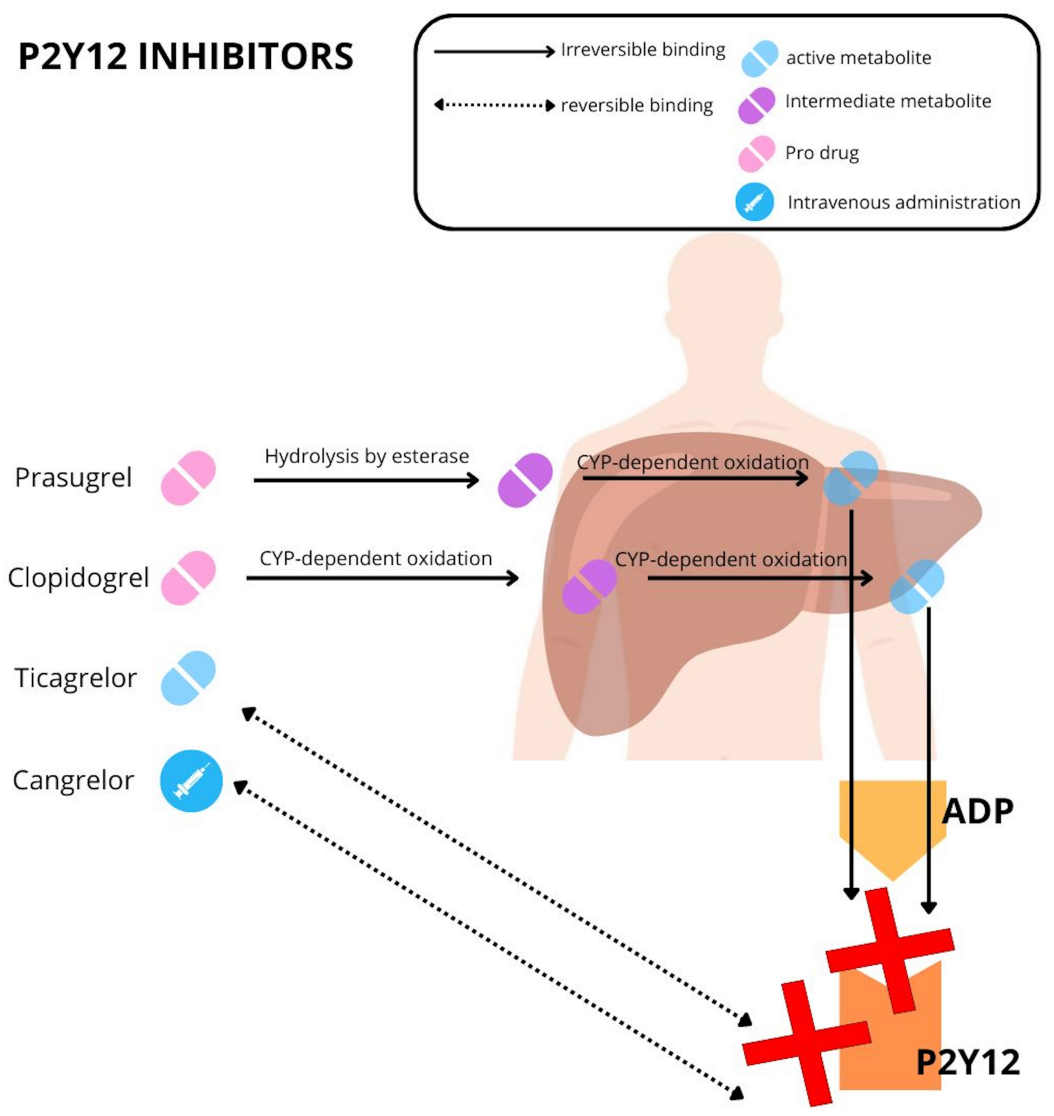
**Mechanism of action of P2Y12 inhibitors**. ADP, adenosine diphosphate; CYP, cytochrome.

An overview of the commercially available P2Y12 inhibitors is summarized in Table 1 and Fig. 2.

**Table 1.** **P2Y12 antagonists**.

	Clopidogrel	Prasugrel	Ticagrelor	Cangrelor
Drug class	Thienopyridine	Thienopyridine	Cyclopentyltriazolo-pyrimidine	Adenosine triphosphate analogue
Reversibility	Irreversible	Irreversible	Reversible	Reversible
P2Y12 receptor interaction	Competitive	Competitive	Allosteric	Competitive
			non-competitive	
Onset of effect	2–6 hours	30 min–4 hours	30 min–2 hours	2 min
Duration of effect	3–10 days	7 days	3–4 days	30–60 min
Delay to surgery	5 days	7 days	5 days	No significant delay
Bioactivation	Yes (pro-drug CYP dependent two step)	Yes (pro-drug CYP dependent, one step)	No	No

CYP, highly polymorphic cytochrome.

### 2.1 Clopidogrel

Clopidogrel, a second-generation oral thienopyridine, permanently inhibits the ADP receptor of blood (P2Y12 receptor). It has replaced ticlopidine, a first-generation thienopyridine, due to its superior safety and comparable efficacy [13].

It is administered as an inactive pro-drug and it needs to be activated via hepatic metabolism by a series of cytochrome P450 enzymes (Fig. 1).

The role of clopidogrel in STEMI was firstly investigated in the Clopidogrel plus Aspirin in Patients with Acute Myocardial Infarction Treated with Fibrinolytic Therapy (CLARITY-TIMI 28) trial, in which 3491 STEMI patients were randomized to receive clopidogrel (300 mg as a starting dose versus placebo). The study resulted in a reduction in the primary endpoint, a composite of cardiovascular (CV) mortality, recurrent myocardial infarction and recurrent ischemia requiring percutaneous coronary intervention (PCI), in patients randomized to the clopidogrel arm (21.7% in the placebo group versus 15.0% in the clopidogrel group; *p* < 0.001), with a comparable incidence of major bleeding according to the TIMI (thrombolysis in myocardial infarction) criteria between the two groups (1.9% in the clopidogrel group versus 1.7% in the placebo group; *p* = 0.80) [14].

Subsequent studies have shown that there are individual differences in the response to the drug that led to inconsistent platelet inhibition and the resulting risk of stent thrombosis and myocardial infarction. These include genetic factors (such as cytochrome P-450 polymorphism), clinical variables (poor absorption, drug interactions), and cellular factors (upregulation of the P2Y12 signaling pathway) [15, 16].

To date, clopidogrel should only be used in patients in whom there is a contraindication to the use of prasugrel or ticagrelor or in whom there is a high risk of bleeding [2].

### 2.2 Prasugrel

Prasugrel is an orally administered third-generation thienopyridine. It acts like an inactive pro-drug but, unlike clopidogrel, requires only a single hepatic oxidation step to produce its active metabolite [17]. Once activated, the metabolite of prasugrel irreversibly binds to the ADP-binding site of the P2Y12 receptor, resulting in greater platelet inhibition than clopidogrel due to its higher plasma concentration [17]. The recommended dose of prasugrel is 10 mg daily, with a loading dose of 60 mg. The Therapeutic Outcomes by Optimizing Platelet Inhibition with Prasugrel Thrombolysis in Myocardial Infarction (TRITON TIMI) 38 study found that prasugrel significantly reduced the primary endpoint (composite endpoint of death, myocardial infarction and stroke) in patients admitted for ACS compared to clopidogrel (9.9% vs 12.1%; hazard ratio, 0.81; 95% confidence interval [CI], 0.73 to 0.90; *p* < 0.001). However, a higher rate of major bleeding was also observed in the prasugrel group (2.4% vs 1.8%; hazard ratio, 1.32; 95% CI, 1.03 to 1.68; *p* = 0.03), particularly in patients over 75 years of age [18]. The available evidence in combination with data from the FAETHER study recommends reducing the dose to 5 mg daily in patients older than 75 years or weighing less than 60 kg; additionally. adverse events associated with Prasugrel are thrombocytopenia, headache and increased liver enzymes. Prasugrel is contraindicated in patients with a history of stroke/transient ischemic attack (TIA) [19].

### 2.3 Ticagrelor

Ticagrelor is a cyclopentyltriazolo-pyrimidine that differs from other thienopyridines and ATP (adenosine triphosphate) analogues. It binds reversibly to the P2Y12 receptor, but at a different site than ADP [20]. Once bound, ticagrelor inhibits the activation of the G protein triggered by ADP binding, keeping the receptor in an inactivated state and preventing ADP signalling [20]. Remarkably, ticagrelor is effective without the need for liver activation. However, about 30% of its effect is due to a metabolite (ARC124910XX) formed by the cytochrome P450 3A4/5 (CYP3A4/5) enzymes. This metabolite has similar pharmacological properties to the parent product [20]. Ticagrelor must be administered twice due to its reversible binding and short half-life. The recommended dosage is 90 mg twice daily, with a loading dose of 180 mg. The onset of action is rapid, between 0.5 and 2 hours, and the duration of action is 3–4 days.

The efficacy of ticagrelor in the treatment of ACS was demonstrated in the PLATO trial, in which ticagrelor showed a reduction in cardiac events compared to clopidogrel in 18,624 patients, who showed a reduction in cardiovascular mortality, myocardial infarction and stroke (9.8% of patients versus 11.7% at 12 months; hazard ratio, 0.84; 95% CI, 0.77 to 0.92; *p* < 0.001) [21]. Of note, there was no significant difference in the bleeding rate between the two groups (11.6% in the ticagrelor group and 11.2% in the clopidogrel group; *p* = 0.43) [21]. The interaction of ticagrelor with adenosine receptors can lead to various adverse effects, such as bradyarrhythmias and dyspnea. The latter may affect 15%–20% of patients.

The ISAREACT 5 trial is the only randomised head-to-head trial to date in which the efficacy of prasugrel and ticagrelor was compared in 4018 patients with ACS [22]. One year after randomization, the incidence of the primary endpoint (composite endpoint of death, myocardial infarction and stroke) was lower in the prasugrel group, mainly due to a lower rate of myocardial infarction (9.1% in the ticagrelor group versus 6.8% in the prasugrel group; hazard ratio, 1.36; 95% CI, 1.09 to 1.70; *p* = 0.006). In addition, the study showed that the benefit of the lower rate of ischemic events was not associated with an increased risk of bleeding (major bleeding was observed in 5.4% of patients in the ticagrelor group and in 4.8% of patients in the prasugrel group [hazard ratio, 1.12; 95% CI, 0.83 to 1.51; *p* = 0.46]). It is important to note that the study compared not only two different drugs, but also two different therapeutic approaches. This study provides an opportunity to re-evaluate the optimal timing for the administration of antiplatelet therapy in patients with non-ST-segment elevation myocardial infarction (NSTEMI). This is because while ticagrelor was administered at the time of randomization, prasugrel was administered only after coronarography, i.e., when coronary anatomy was known [22]. Currently, the role of pretreatment is controversial, especially in STEMI, and has been the subject of several studies.

### 2.4 Cangrelor

Cangrelor is a fast-acting intravenous P2Y12 antagonist that belongs to the family of ATP analogues. The site of binding to the P2Y12 receptor has not yet been identified. Nevertheless, cangrelor is highly effective in inhibiting ADP binding and inducing rapid platelet inhibition. Ectonucleotidase-mediated dephosphorylation serves to inactivate the drug, which has a short half-life of 3–6 minutes, allowing rapid recovery of platelet function (60 minutes) [23].

The recommended dosage is 30 mcg/kg bolus followed by a continuous infusion of 4 mcg/kg/min without the need for dose adjustment based on age, renal or hepatic function. Cangrelor has been studied in three different Phase 3 trials, but only Champion Phoenix showed superiority over clopidogrel in reducing the primary endpoint, a composite of all-cause death, myocardial infarction, ischemia-driven revascularization (IDR) or stent thrombosis 48 hours after randomization [24, 25, 26].

A total of 11,145 patients were randomly assigned to either clopidogrel or cangrelor. A total of 56.1% of patients had stable angina, 25.7% had NSTEMI and 18.2% had STEMI. The cangrelor group had a significantly lower primary endpoint rate (4.7% in the cangrelor group versus 5.9% in the clopidogrel group, OR 0.78, a 95% CI of 0.66 to 0.93, *p* = 0.005) [26]. Consistent with previous findings, a meta-analysis of data from all three studies showed that cangrelor is associated with a lower combined rate of adverse events without an increase in major bleeding [27].

## 3. P2Y12 Inhibitors Pretreatment in STEMI

Table 2 (Ref. [28, 29, 30]) summarizes the main features of the studies mentioned below.

**Table 2.** **Summary of the main pre-treatment studies**.

Characteristics	Atlantic trial [30]	Redfors *et al*. [28]	Rohla *et al*. [29]
Setting	STEMI	STEMI	STEMI
Trial design	Phase 4, randomized, doble-blind	Non-randomized, observational	Non-randomized, observational
N. of enrolled patients	1862	44,804	1996
Pre-treatment	Ticagrelor 180 mg pre hospital vs ticagrelor 180 mg in hospital	Pre-treatment with prasugrel or ticagrelor or clopidogrel (57.2%)	Prasugrel and ticagrelor was preferred
Key time delays	159 min between symptom onset and pPCI, 31 min between the loading dose and pPCI	189 min between symptom onset and pPCI; unaware of the time difference in loading dose and pPCI	220 min between symptom onset and pPCI 62 min between loading dose and pPCI
Primary study endpoint	Two co-primary (surrogate) endpoints: proportion of patients	Mortality at 30 days	Composite end point all-cause death, recurrent MI, stroke, or definite stent thrombosis
	without ≥70% resolution of ST-segment elevation before PCI; the proportion of patients without TIMI III flow in infarct-related artery		
Key bleeding endpoint	Non-CABG-related bleeding	In hospital bleeding	BARC type 3 or 5 bleeding
Glycoprotein IIb/IIIa	Administered to approximately one-third of patients	Allowed, no differences between groups	Allowed, no differences between groups

BARC, bleeding academic research consortium; pPCI, primary percutaneous coronary intervention; STEMI, ST-elevation myocardial infarction; TIMI, thrombolysis in myocardial infarction; MI, myocardial infarction; CABG, coronary artery bypass grafting.

In interventional cardiology, the term pretreatment is used to describe a therapeutic strategy in which the P2Y12 inhibitor is administered prior to coronary angiography.

This strategy hypothetically offers several advantages, such as (a) sufficient time for the orally administered drug to exert its full antiplatelet effect, (b) protection from ischemia while waiting for coronary angiography, (c) reduction in the risk of periprocedural thrombotic complication, (d) reduction in the need for glycoprotein IIb/IIIa bailout.

**Fig. 3.**
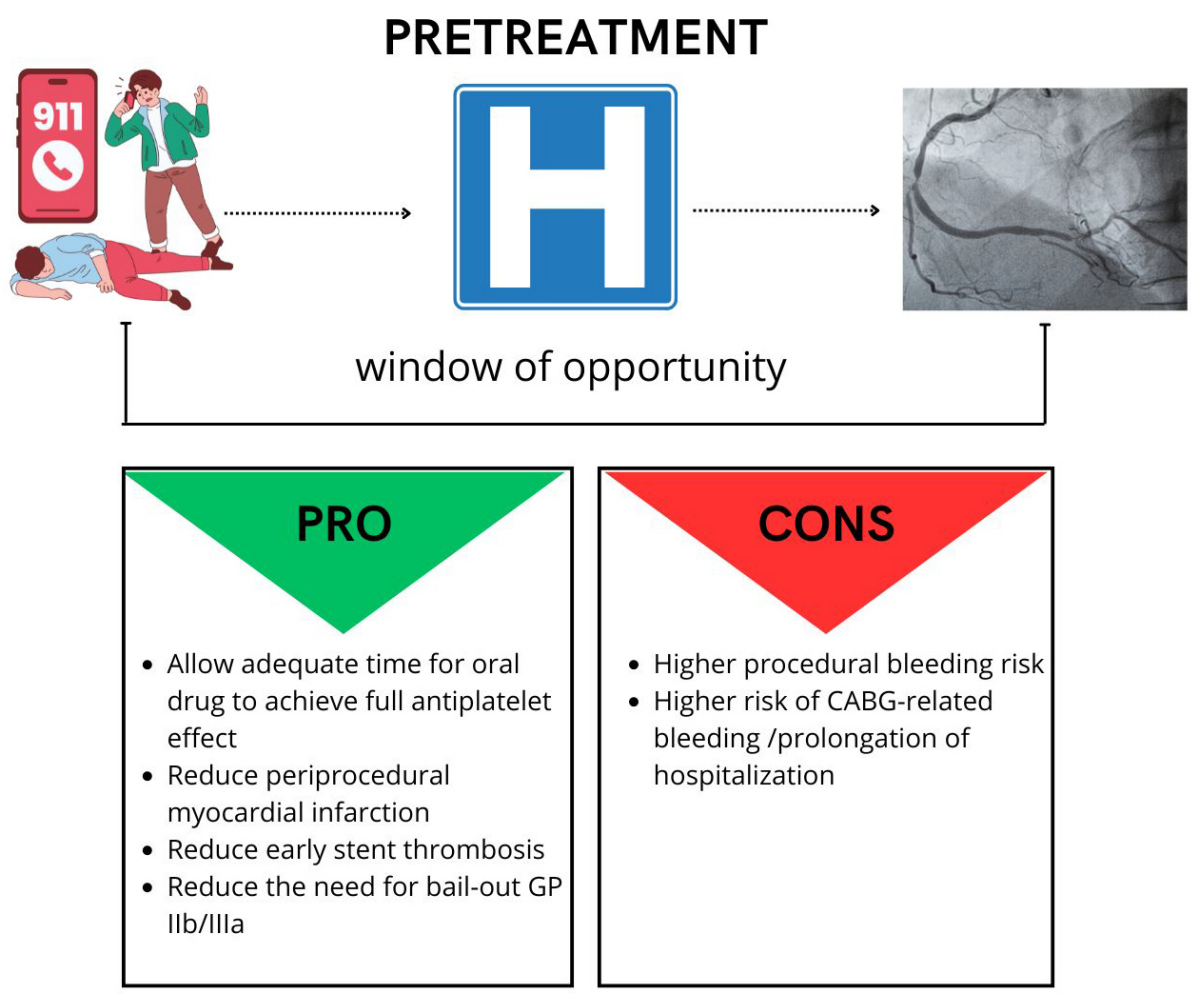
**Pretreatment pro and cons**. CABG, coronary artery bypass grafting.

On the other hand, it may increase bleeding risk and delay the procedure in patients who are candidates for coronary artery bypass grafting (CABG) (Fig. 3). However, while the rate of surgical revascularization in NSTEMI is not trivial, patients with STEMI are usually referred for percutaneous revascularization.

It appears that pre-treatment in STEMI patients may be appealing due to the underlying pathophysiology. STEMI patients have highly activated platelets and ruptured plaques with a thrombus is usually present in at least one coronary artery [31].

High platelet reactivity has been shown to correlate with the extent of myocardial damage, suboptimal flow in the infarct-related artery after percutaneous coronary intervention and the extent of microvascular obstruction [32, 33, 34]. Therefore, early inhibition of platelets with P2Y12 inhibitors could have a protective effect against further myocardial injury and microvascular obstruction and reduce ischemic complications.

In the current European guidelines, pretreatment can be considered for patients with STEMI undergoing a primary PCI strategy (Class IIb Level of Evidence B) [2].

However, this recommendation is only supported by a limited amount of evidence.

The initial evidence supporting pre-treatment stems from PCI-CURE (clopidogrel in unstable angina to prevent recurrent events), demonstrating that pre-treatment with clopidogrel reduced the incidence of cardiovascular adverse events in non ST elevation acute coronary syndrome (NSTEACS) patients eligible for PCI. However, the mean interval between pre-treatment and the invasive procedure was approximately 6 days, a timeframe that deviates from current guidelines [1].

In 2012, Zeymer *et al*. [35] conducted a study to evaluate the efficacy of pretreatment with clopidogrel (loading dose (LD) 600 mg). Out of the 337 randomized patients, 166 received a loading dose before coronarography. The study found no significant difference in TIMI 2/3 patency rate before PCI between the two groups (49.3% vs 45.1%, *p* = 0.5). However, there was a noticeable trend indicating a potential reduction in the combined endpoint of death, re-infarction, and urgent target vessel revascularization in the prehospital-treated patients (3.0% vs 7.0%, *p* = 0.09).

Additionally, the pretreatment strategy was found to be safe without an increasing major bleeding [35].

In 2019, Redfors *et al*. [28] analyzed a large observational cohort of patients from the Swedish Coronary Angiography and Angioplasty Registry (SCAAR). The study collected data from patients who had undergone primary angioplasty between 2005 and 2016 and compared patients who were receiving a P2Y12 inhibitor at the time of first medical contact with those who were receiving a P2Y12 antagonist at the time of PCI. The authors found no statistically significant difference in 30-day mortality (5.2% vs 7.6%, *p* = 0.313), stent thrombosis (0.6% vs 0.6%, *p* = 0.932) or increased bleeding risk (2.6% vs 3.4%, *p* = 0210) between the group with pretreatment and the group without, suggesting that the administration of P2Y12 inhibitors during pPCI (primary percutaneous intervention) have no significant impact on patient outcomes. However, caution is required when analysing the results. First, clopidogrel was the most commonly used P2Y12 inhibitor (in 58% of cases), and its slow onset of action may have compromised treatment efficacy. Second, it should be noted that we do not currently know the time interval at which antiplatelet agents were administered between groups, which may have implications for the interpretation of the data. Third, the differences in baseline characteristics between groups are substantial (e.g., pre-treated patients were younger and less likely to have major cardiovascular risk factors, heart failure, previous myocardial infarction or complex coronary artery disease).

The present results are consistent with those of Koul *et al*. [36]. 7433 patients were included in the analysis, of whom 5438 received pretreatment and 1995 received ticagrelor in the catheterization laboratory. Pretreatment ticagrelor administration did not improve the composite primary endpoint (6.2% vs 6.5%, *p* = 0.69) or its individual components, characterized by all-cause mortality (4.5% vs 4.7% *p* = 0.86), myocardial infarction (1.6% vs 1.7% *p* = 0.72), and stent thrombosis (0.5% vs 0.4% *p* = 0.80), at 30 days compared with ticagrelor given in the catheterization laboratory [36].

A recent study by Rohla *et al*. [29] examined a group of patients from the Bern Registry and stratified them into two cohorts based on the recommended P2Y12 inhibitor administration strategy (pre-treatment vs non-pre-treatment). The study included a total of 1506 participants, of which 708 patients received pre-treatment, while 798 were not treated. The study found no statistically significant differences between the two treatment strategies in terms of major adverse cardiac and cerebrovascular events (MACCEs), all-cause mortality, recurrent myocardial infarction, stroke or 30-day stent thrombosis (7.1% vs 8.4%; adjusted HR: 1.17; 95% CI: 0.78–1.74; *p* = 0.45), and pre-treatment was shown to be a safe strategy with no increased risk of bleeding (3% vs 3.3% adjusted HR 0.82 (0.43–1.54, *p* = 0.53). Ticagrelor was the most commonly used P2Y12 inhibitor in both the pretreated and non-pretreated groups, with use rates of 86% and 72.7%, respectively, while prasugrel and clopidogrel were used in 12% and 8% of cases, respectively. However, as this was an observational study, some limitations must be pointed out. The two cohorts differed in terms of ischemia time and clinical presentation. In particular, the pre-treatment cohort had a longer ischemia time and was less likely to have cardiogenic shock.

The Atlantic trial [30] is currently the only randomized study to report a benefit of pre-treatment. This trial involved 1875 patients who were randomized to receive a 180 mg loading dose of ticagrelor either before or during hospital treatment. While there was no statistically significant difference in the primary endpoint, there was a reduction in stent thrombosis at both 24 hours and 30 days in the prehospital group (0% at 24 hours in the prehospital group versus 0.8% in the in-hospital group *p* = 0.008 and at 30 days 0.2% vs 1.2%, *p* = 0.02).

While the decrease in stent thrombosis in the prehospital group is a notable finding, it is interesting to note that no other primary or secondary endpoints were associated with it; on the contrary, a trend towards increased mortality, despite not significant, was observed in the prehospital group. Common risks associated with prehospital administration of P2Y12 inhibitors include bleeding and possible delays in any necessary bypass surgery. However, none of these events occurred in the Atlantic trial. This could be due to the low CABG rate (<2%) and the almost exclusive use of radial access. In addition, the 31-minute time difference between the administration of ticagrelor in the two groups could also be a confounding factor. Due to the short time difference, it is difficult to expect a pre-treatment benefit.

The aforementioned hypothesis is supported by a recent study of Almendro-Delia *et al*. [37]. Among 1624 patients analyzed, 1033 received the P2Y12 inhibitor before coronarography. The study revealed that the primary endpoint, a composite of all-cause death, recurrent myocardial infarction, stroke, urgent target lesion revascularization, or definite stent thrombosis, was statistically superior in the non-pretreated group (11% vs 7.5%, *p* < 0.001). Additionally, the authors observed a time-dependent relationship of pre-treatment revealing that the overall advantage of early versus delayed P2Y12 inhibition only becomes apparent after at least 80 minutes of loading dose administration. The relationship observed exhibits a U-shaped pattern, indicating that the most significant advantage lies within the middle portion of the curve. At both extremities, pre-treatment does not confer any benefits; on one hand, the drug is unable to take effect, and on the other, it delays pPCI [37].

Even Pepe *et al*. [38] also noted a time-dependent effect, indicating that patients who received pre-treatment at least one hour before PCI exhibited an improved time flow grade before pPCI and had a higher likelihood of achieving a TFG (Thrombolysis in Myocardial Infarction flow grade) 3 after pPCI. Addionally, the adverse events occurring during 30-day follow-up period may be less directly associated with the initial treatment, thereby concealing the positive impacts of pre-treatment. The Atlantic H 24, a sub study of the Atlantic trial, investigated the effects of pre-treatment within the initial 24 hours. Despite no variance in reperfusion before PCI, the authors highlighted a statistically significant difference between the two groups in terms of the composite endpoint of death, new MI, urgent revascularization, definite stent thrombosis, bail-out glycoprotein IIb/IIIa inhibitor use (10.4% vs 13.7%, *p* = 0.039). Notably, the pre-hospitalization group exhibited a remarkable advantage concerning both stent thrombosis (0% vs 1%, *p* = 0.008) and new MI (0% vs 0.7%, *p* = 0.031) [39].

The rapid time-to-balloon required for STEMI has limited the window of opportunity for pre-treatment. In addition, the effect of P2Y12 inhibitors is often delayed in STEMI patients due to reduced cardiac output and vasoconstriction of peripheral arteries, resulting in shunting of blood to vital organs and reduced drug absorption [40, 41]. In addition, frequent nausea and vomiting in the acute setting and the use of morphine or opioid analgesics for pain relief can hinder drug absorption by slowing intestinal motility [42]. It is worth noting that increasing the loading dose does not resolve this problem, and there is limited evidence on the use of oro-dispersible formulations or crushed tablets [43, 44, 45, 46]. In the Load and Go trial the authors compared prehospital administration of two dosages of clopidogrel, either 600 mg or 900 mg, with the periprocedural use of 300 mg of clopidogrel in patients with STEMI undergoing primary PCI. Notably, the primary endpoint, defined as achieving thrombolysis in myocardial infarction perfusion grade 3 (TMPG 3), did not exhibit significant differences (TMPG 3 was 64.9% for pretreatment with either 600 mg or 900 mg vs 66.1% in the no-pretreatment arm *p* = 0.88) [47].

An alternative to pre-treatment could be the use of cangrelor, an intravenous P2Y12 antagonist with rapid onset and offset of action. However, there are currently no data comparing its efficacy with other P2Y12 inhibitors such as ticagrelor or prasugrel [25, 26].

The study that first showed a positive effect of cangrelor in the setting of PCI was the Effect of Platelet Inhibition with Cangrelor during PCI on Ischemic Events (CHAMPION PHOENIX) study. Although CHAMPION PHOENIX was not a pre-treatment study, it showed that stronger platelet inhibition at the time of PCI can have a positive impact on ischemic endpoints. Although cangrelor was expected to provide greater benefit in patients diagnosed with STEMI, the benefit observed in Champion Phoenix was the same in all groups (STEMI, NSTEMI, unstable angina; *p* = 0.98). In addition, it is important to emphasize that only 63% of patients received clopidogrel before PCI and that no information is available on the time interval between drug administration and PCI [26].

In the platelet inhibition with cangrelor and crushed ticagrelor in STEMI patients undergoing primary percutaneous coronary intervention (CANTIC) study, the efficacy of crushed ticagrelor plus cangrelor during primary PCI was compared with crushed ticagrelor plus placebo. The study showed that platelet reactivity was significantly lower in the cangrelor group than in the placebo group after 30 minutes (P2Y12 reaction unit [PRU] 63 [32–93] vs 214 [183–245] *p* < 0.001) and until the end of the infusion [48]. Furthermore, platelet reactivity was the same in both groups two hours after the end of the infusion, suggesting that there was no interaction between the two drugs. Whether this reduction in platelet activity has a clinical benefit is not yet clear. On the other hand, in the Fabolous-Faster study, it was found that 30 minutes after the start of infusion, platelet activity was higher in the cangrelor group than in the tirofiban group (percentage of inhibition of platelet aggregation [IPA%] 95.0 ± 9% in the tirofiban group versus 34.1 ± 22.5% in the cangrelor group *p* < 0.001), with cangrelor providing better inhibition of platelet aggregation compared to prasugrel at 30 minutes (platelet aggregation (PA)% 34.1 ± 22.5% vs 10.472 ± 11.040% *p* < 0.001) [49].

In a recent study by Ubaid *et al*. [50], 100 patients with STEMI were randomly assigned to either cangrelor or ticagrelor. The cangrelor infusion was initiated immediately after randomization, and a loading dose of ticagrelor was administered 30 minutes before the end of the infusion. Although the researchers found greater platelet inhibition in the cangrelor group during primary PCI (*p* < 0.0001), there was no statistically significant difference in secondary endpoints such as microvascular resistance (*p* = 0.52) and final infarct size (*p* = 0.61) [50].

So far, cangrelor has been identified as a potential therapeutic option for certain patient groups, e.g., patients with cardiogenic shock, post-cardiac arrest, or intubated patients. As previously mentioned, the presence of peripheral arterial vasoconstriction, vomiting or opioid use can reduce the intestinal absorption of oral P2Y12 inhibitors [47, 51].

Another potential application of cangrelor is in patients who have not previously been treated with P2Y12 inhibitors and who require complex angioplasty, i.e., left main or true bifurcations. In such cases, the rapid inhibition of platelet activity by cangrelor could reduce the risk of thrombotic complications.

Further P2Y12 inhibitors are currently in development [52].

## 4. Selection of P2Y12 Antagonists in Different Clinical Scenarios

In the management of patients diagnosed with STEMI, the recommended therapeutic approach involves the administration of dual antiplatelet therapy. The selection of the P2Y12 inhibitor necessitates a comprehensive evaluation of both thrombotic and hemorrhagic risks, with the latter being determined using criteria outlined by the Academic Research Consortium. In cases where there are no contraindications or a low risk of hemorrhage, current guidelines advocate for the utilization of ticagrelor or prasugrel over clopidogrel. The latter is preferable for patients with a history of hemorrhagic stroke, those on long-term oral anticoagulant therapy, or individuals with moderate-to-severe liver disease.

It is imperative to exercise particular vigilance in the elderly population, where the presence of comorbidities not only heightens the risk of ischemia but also exacerbates the susceptibility to hemorrhage.

In situations where oral medication is not feasible, clinicians should contemplate the utilization of cangrelor. This is notably applicable to intubated patients who have suffered a cardiac arrest outside of the hospital, and to patients experiencing severe nausea. Furthermore, in patients with cardiogenic shock, a low flow rate may lead to decreased bioavailability of oral antiplatelets, thus making it necessary to consider cangrelor as an alternative to mitigate the risk of thrombosis.

## 5. Future Prespective 

The scientific evidence in favour of the use of pretreatment in STEMI is currently limited. In addition, the need for early invasive treatment limits the window of opportunity, leaving less time for the potential beneficial effects. Furthermore, with the introduction of cangrelor, the need for pretreatment may become obsolete.

However, cangrelor can only be administered intravenously by healthcare professionals, so the development of an easy-to-administer, fast-acting drug could be useful.

It may be worth considering selatogrel as a reversible P2Y12 inhibitor as it is fast acting and can be administered subcutaneously [53, 54].

Interestingly, the drug is mainly excreted via the faeces, so that renal function is less of a concern [55]. However, caution should be exercised in patients with moderate liver dysfunction. Although subcutaneous administration allows for self-administration, it can be difficult for patients to recognize the symptoms of an infarction, even if they have experienced one previously. Fortunately, phase 2 trials have shown a favourable risk profile for the drug, with injection site bruising being the main bleeding event [56]. Further information on the efficacy of the drug is likely to emerge from the ongoing Selatogrel Outcome Study in Suspected Acute Myocardial Infarction (SOS-AMI) trial.

It is important to consider that patients with STEMI may have different clinical features such as age, gender and the localization of ST-segment elevation on electrocardiography, which may indicate the presence of single or multivessel disease. These factors may influence the decision and benefit of pre-treatment. Therefore, a personalized approach based on the unique characteristics of the patient may be recommended. However, it is crucial to note that accurately assessing the patient for STEMI can be challenging and that any assessment should not delay the time between first medical contact and pPCI [57].

**Fig. 4.**
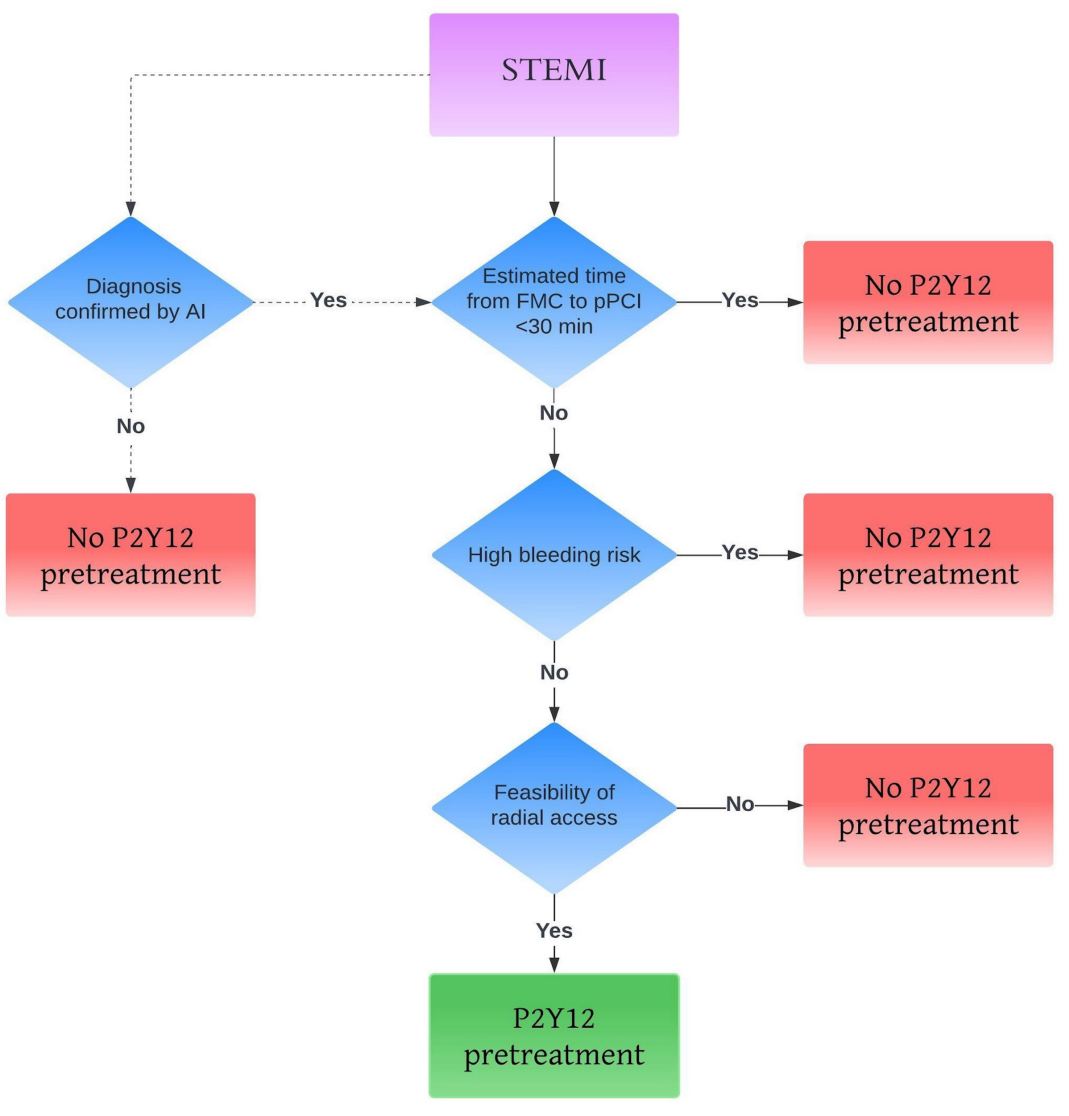
**Personalized approach for pretreatment**. The dotted line represents the potential use of artificial intelligence (AI). STEMI, ST elevation myocardial infarction; pPCI, primary percutaneous coronary intervention; FMC, first medical contact.

Fig. 4 proposes an algorithm for selecting patients who may benefit from pretreatment. Clinicians should consider several factors, such as the estimated time between first medical contact and PCI, the risk of bleeding and the feasibility of radial access [58, 59, 60, 61]. The dotted line in the figure also shows the potential use of artificial intelligence (AI).

AI could be helpful in the accurate diagnosis of STEMI, especially for non-cardiologists, reducing false positives and enabling early administration of P2Y12 [62].

## 6. Conclusions 

In the contemporary management of STEMI, pretreatment with P2Y12 inhibitors remains controversial due to its potential benefits and risks. While pretreatment may offer benefits such as increased platelet inhibition, reduced thrombotic complications and improved microvascular outcomes, it also carries the risk of increased bleeding and the possibility of delaying procedures such as coronary artery bypass grafting.

The evolving landscape of interventional cardiology suggests that the future of pretreatment may be shifting towards fast-acting, easy-to-administer therapies that can provide rapid platelet inhibition without the limitations of oral P2Y12 inhibitors. Personalized treatment approaches that consider individual patient characteristics such as bleeding risk, ischemic burden and procedural timing are likely to become increasingly important in optimizing outcomes for STEMI patients.

## Abbreviations

%IPA, percentage of inhibition of platelet aggregation; ACS, acute coronary syndrome; ADP, adenosine diphosphate; AI, artificial intelligence; ATP, adenosine triphosphate; CABG, coronary artery bypass grafting; CANTIC, platelet inhibition with cangrelor and crushed ticagrelor in STEMI patients undergoing primary percutaneous coronary intervention; CHAMPION PHOENIX, Effect of Platelet Inhibition with Cangrelor during PCI on Ischemic Events; CURE, clopidogrel in unstable angina to prevent recurrent events; DES, drug-eluting stent; FAETHER, Comparison of Prasugrel and Clopidogrel in Low Body Weight Versus Higher Body Weight With Coronary Artery Disease; CV, cardiovascular mortality; CVD, cardiovascular disease; DAPT, dual antiplatelet therapy; IDR, ischemia-driven revascularization; MACCEs, major cardiac and cerebrovascular adverse events; MI, myocardial infarction; NSTEMI, non-ST segment elevation myocardial infarction; P13K, P13 kinase; PRU, P2Y12 reaction unit; PCI, percutaneous coronary intervention; PLATO, Platelet Inhibition and Patient Outcomes; pPCI, primary percutaneous intervention; SCAAR, Swedish Coronary Angiography and Angioplasty Registry; STEMI, ST elevation myocardial infarction; TRITON TIMI 38, Therapeutic Outcomes by Optimizing Platelet Inhibition with Prasugrel–Thrombolysis in Myocardial Infarction; TXA2, thromboxane A2; TIMI, thrombolysis in myocardial infarction.
